# *Chapelieria magna*, a new species of Rubiaceae from eastern Madagascar

**DOI:** 10.3897/phytokeys.44.8513

**Published:** 2015-01-15

**Authors:** Kent Kainulainen, Sylvain G. Razafimandimbison

**Affiliations:** 1 The Bergius Foundation at the Royal Swedish Academy of Sciences; 2 Department of Ecology, Environment and Plant Sciences, Stockholm University, SE-106 91, Stockholm, Sweden

**Keywords:** *Chapelieria*, Madagascar, Octotropideae, Rubiaceae

## Abstract

A new species of *Chapelieria* was discovered during a recent field trip to the Masoala National Park in eastern Madagascar, and is described here as *Chapelieria magna* Kainul., **sp. nov.** This species is readily distinguishable from previously described species of the genus by its quadrangular shoots, triangular-calyptrate stipules, sessile leaves, pubescent styles, and ridged fruits. It also differs in the larger number of ovules and the much larger size of leaves and fruits.

## Introduction

*Chapelieria* A. Rich., is a genus endemic to Madagascar that belongs to tribe Octotropideae (Rubiaceae; subfamily Ixoroideae). The taxonomic history of the genus is complex (Madagascar Catalogue 2014). *Chapelieria madagascariensis* A. Rich. was originally described by Richard (1830), commemorating Louis Armand Chapelier who had collected the type material in eastern Madagascar. However, the name was first published by De Candolle in September 1830, citing Richard’s (1830) manuscript that was not published until December that same year (Stearn 1957). De Candolle’s (1830) description is essentially identical, but with an added note on the similarity of the plant habit to that of an Apocynaceae. Baillon (1880), considered *Chapelieria* and *Tamatavia* Hook.f. as congeneric. *Tamatavea melleri* had been described by Hooker (1871: pl 1090) just a few years earlier although with some reservation as to its novelty: “I advance this genus as new with some hesitation, because it may prove to be one of the several Madagascarian genera which are so imperfectly or incorrectly characterized in systematic works, that it is impossible to recognize them by their description”. Schumann (1891), included *Tamatavia melleri* in *Chapelieria* as *Chapelieria melleri*, and Chevalier (1942) subsequently synonymized the two names.

In a revision of Malagasy Apocynaceae, Pichon (1949) noted that the type material of *Chapelieria madagascariensis* was mixed and included both Apocynaceae and Rubiaceae material, and consequently he synonymized the name under *Carissa edulis* Vahl *var. septentrionalis* Pichon. The Apocynaceae specialists Markgraf (1976), and Leeuwenberg and van Dilst (2001), also considered *Chapelieria* a synonym of *Carissa*. Recently, however, Davies and Davis (2014), emended the description of *Chapelieria madagascariensis* and specified one of the Chapelier specimens as the holotype (a paper to clarify the issue of the typification is in preparation, Davis AP, pers. comm.). They also described two new species of *Chapelieria* (*Chapelieria multiflora* N.M.J. Davies & A.P. Davis and *Chapelieria septentrionalis* N.M.J. Davies & A.P. Davis), and estimated the total number of species in Madagascar to be about ten.

During a recent field trip to southern Masoala National Park, we collected an unknown *Chapelieria* and it is here described as a new species. Morphologically, the plant conforms to the characterization of tribe Octotropideae by Tosh et al. (2009), having articulated petiole bases with distinct sutures, paired supra-axillary inflorescences, hermaphroditic flowers with secondary pollen presentation, funnelform corollas with left-contorted aestivation, 2-locular ovaries with axile placentation, pendulous ovules, and striate pattern of the seed coat. Characters that support a placement in *Chapelieria* as described by Davies and Davis (2014), include the sessile inflorescences, sessile flowers, 5-merous flowers, and seeds with entire endosperm. In contrast, the new species does not have grooved/ridged styles, and further broadens the generic description of *Chapelieria* (Davies and Davis 2014) by having stipules fused to a cap that cover the apical buds, sessile leaves, simple styles (not club-shaped), and in the larger number of ovules per locule (16 vs. 3–7). Furthermore, the styles of this species are sparsely pubescent, and the fruits are distinctly ribbed. The latter two traits are also be found in the genus *Flagenium* Baill. Characters that distinguish *Flagenium* from *Chapelieria* include the presence of both erect and pendulous ovules, and the absence of articulated petioles (Ruhsam and Davis 2007). Preliminary molecular phylogenetic analyses of both cpDNA and rDNA data support a position of the new species in *Chapelieria* (Kainulainen et al. unpublished data).

Flower buds of *Chapelieria* are enclosed by calyptrate bracts (Chevalier 1947), and this is also the case in *Chapelieria magna*. The conical sheath formed by the fused bracts is split by the expanding flower buds, but the bracts persist as an asymmetric triangular sheath around the inflorescence branches. Lateral buds appear to form continuously, and many buds of varying levels of development are found within the cymose inflorescences. However, because of the congested nature of the inflorescence, branchlets with primordial buds may appear as single bracteolate flowers.

## Taxonomy

### Chapelieria magna

Taxon classificationPlantaeGentianalesRubiaceae

Kainul. sp. nov.

urn:lsid:ipni.org:names:77144550-1

Figures 1 , 2

#### Diagnosis.

Differs from previously described species of *Chapelieria* (*Chapelieria madagascariensis*, *Chapelieria multiflora*, and *Chapelieria septentrionalis*) by its quadrangular shoots; triangular-calyptrate stipules; sessile leaves (vs. petiole 5–11 mm); simple, terete, sparsely pubescent styles (vs. club-shaped, grooved/ridged, glabrous styles); ovule number (ca. 16 vs. 3–7 per locule); distinctly ridged fruits (vs. ±smooth fruits); and the much larger size of leaves (up to 42 × 12.2 cm vs. <16.6 × 7.8 cm), and fruits (up to 45 × 20 mm vs. <13 × 7.0 mm).

#### Type.

**MADAGASCAR.** Toamasina Province: Analanjirofo Region, Maroantsetra District, Masoala National Park, 15°41.910'S; 49°57.815'E, 115 m altitude, 15 January 2013 (fl.), *S.G. Razafimandimbison et al. 1240* (holotype S!, isotype, TAN!).

#### Description.

Treelet, to 4 m tall, all vegetative parts glabrous; with decussate, horizontal branches; branchlets quadrangular, 4.0–7.0 mm in diameter, bark drying brown. Stipules ca. 25–30 mm long, initially calyptrate and covering the apical bud, subsequently interpetiolar, triangular, with raised median line and apiculate apex; persistent. Leaves: sessile, narrowly obovate, ca. 39.0–42.0 × 10.5–12.2 cm; bases acute–auriculate; apices acute; adaxial surface: green when fresh, drying pale brownish-gray, smooth, secondary veins brochidodromus, obvious, curved, 15–20 pairs; midribs prominent, pale green when fresh, ±the same colour of the leaf when dry; abaxial surface: pale green when fresh, pale brown when dry, veins reddish-brown. Inflorescences ±sessile, many-flowered (although only 1–few flowers may be mature at any given time); bracts initially calyptrate and covering the flower buds, subsequently splitting unequally to asymmetric, ±triangular sheaths, ca. 18 × 21 mm (1^st^ order bracts), pale green–bright reddish pink, adaxially glabrous, abaxially densely strigose (hairs ca. 0.9 mm), bracteoles reduced; Flowers: hypanthium narrowly urceolate, ca. 6.7 × 2.0 mm; calyces greenish white–bright reddish pink; calyx tubes 3.0–5.0 mm long, externally glabrous, but with hairs (ca. 0.5–1.0 mm long) and colleters on the lower inner surface; calyx lobes ca. 7.5 × 1.3 mm, narrowly triangular, with ciliolate margins (hairs ca. 0.5–2.5 mm); corollas white, funnelform, ±curved; corolla tubes ca. 15 mm long, externally and internally glabrous; corolla lobes ca. 10 × 4.6 mm long, acute, recurved at anthesis; stamens: sessile, attached ca. 3 mm below corolla sinus; anthers white, ca. 7.9 × 0.8 mm, linear, medifixed, exserted for ca. 0.5–1.0 mm; styles simple, ca. 16.5 mm long, sparsely pubescent (hairs ca. 0.5 mm long); stigmas shortly bifid (lobes ca. 0.5 mm long); exserted for ca. 0.5–1.0 mm; ovary ellipsoid, 2-locular, ovules arranged in two series, pendulous, ca. 16 ovules per locule; Fruits: mature fruits red, ca. 36–45 × 14–20 mm, glabrous, fleshy-indehiscent, fusiform, and apically elongated, with distinctive longitudinal grooves/ridges; calyx lobes persistent. Seeds: maturing at ± same rate, ca. 4.8–6.8 × 4.0–6.0 mm, compressed and angular.

#### Distribution and habitat.

*Chapelieria magna* is only known from the type collection, made from a small stand of understory treelets in the rainforest of southern Masoala National Park. Notably, *Chapelieria madagascariensis* also occurs in this area. Although previously only known from the (eastern) Masoala peninsula by a collection made in 1951 (A. Tata 3404-RN; Davies and Davis 2014), we collected a specimen 4.7 km south of the *Chapelieria magna* locality in the nearby Tampolo littoral forest (*Razafimandimbison et al. 1217A*; S, TAN). However, whereas *Chapelieria madagascariensis* was found on sandy soil (cf. Davies and Davis 2014), the habitat of *Chapelieria magna* was on lateritic soil.

#### Phenology.

Both flowers and fruits were found when we collected *Chapelieria magna* in mid-January. This is during the rainy season in Madagascar.

**Figure 1.**
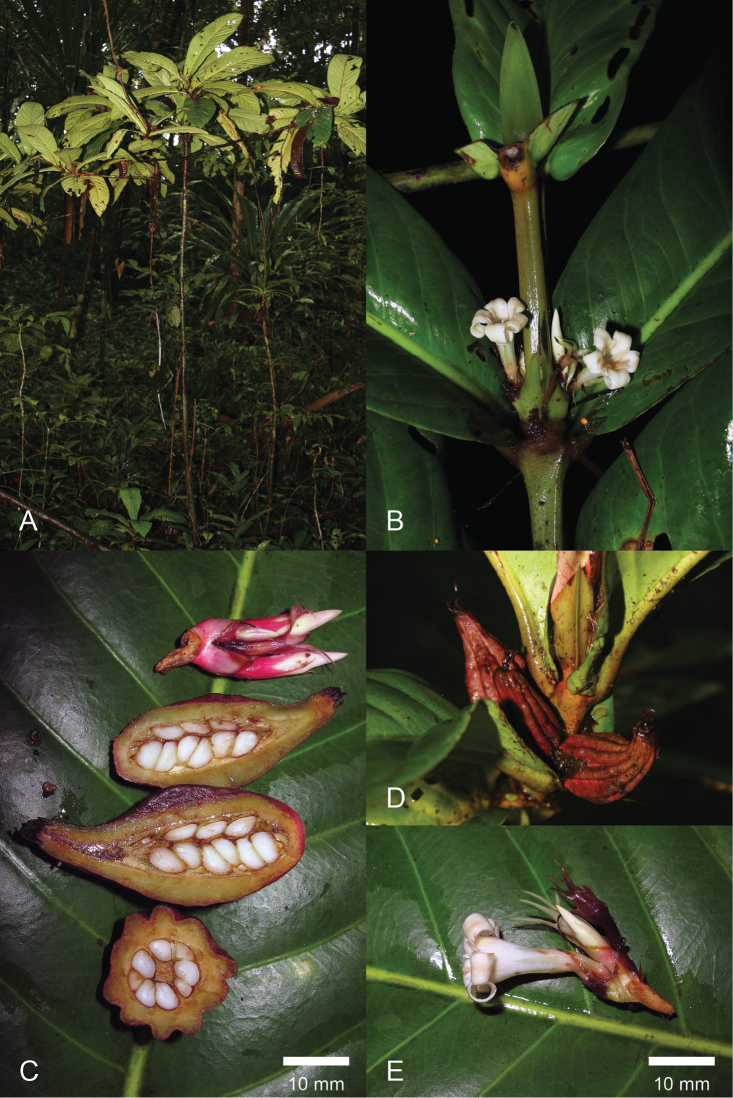
*Chapelieria magna*. **A** Habit and habitat **B** Flowering branch. Note the apical calyptrate stipules (one leaf removed) **C** Flower buds, and fruits in longitudinal and transversal sections, on leaf (×1.5) **D** Fruits **E** Inflorescence on leaf (×1.5). Photographs by Kent Kainulainen.

**Figure 2.**
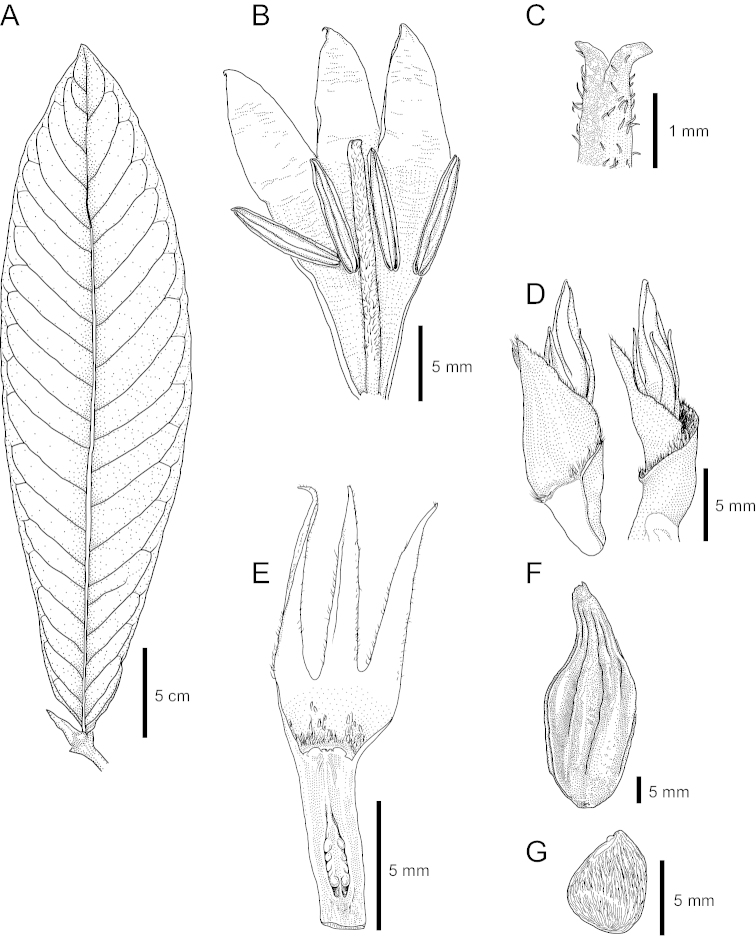
*Chapelieria magna*. **A** Leaf **B** Longitudinal section of corolla. **C** Stigma **D** Part of an inflorescence with flower buds and bracts **E** Longitudinal section of ovary and calyx **F** Fruit **G** Seed. Drawings from the holotype: *Razafimandimbison et al. 1240*, by Kent Kainulainen.

## Supplementary Material

XML Treatment for Chapelieria magna

## References

[B1] Baillon HE (1880) Histoire des plantes 7. Hachette, Paris, 1–546.

[B2] Chevalier AJB (1942) Les caféiers du globe, fasc. 2: iconographie des caféiers sauvages et cultivés et des Rubiacées prises pour des caféiers. Encyclopédie Biologique 22: 1–36.

[B3] Chevalier AJB (1947) Les caféiers du globe, fasc. 3: systématique des caféiers et faux caféiers, maladies et insectes nuisibles. Encyclopédie Biologique 28: 1–356.

[B4] Davies NMJ Davis AP (2014) *Chapelieria septentrionalis* and *C. multiflora* spp. nov. (Rubiaceae, Octotropideae) and an emended description for *C. madagascariensis*. Nordic Journal of Botany 32(6): 691–700. doi: 10.1111/njb.00459

[B5] De Candolle AP (1830) Rubiaceae. Prodromus systematis naturalis regni vegetabilis 4. Treuttel & Würtz, Paris, 341–622.

[B6] Hooker JD (1871) *Tamatavea melleri*. Hooker’s Icones Plantarum 11: pl. 1090.

[B7] Leeuwenberg AJM van Dilst FJH (2001) Series of revisions of Apocyanaceae XLIX, *Carissa* L. Wageningen University Papers 1: 3–109.

[B8] Madagascar Catalogue (2014) Catalogue of the vascular plants of Madagascar. Missouri Botanical Garden, St. Louis, U.S.A. & Antananarivo, Madagascar http://www.efloras.org/madagascar [accessed: 08.2014]

[B9] Markgraf F (1976) Apocynaceae. In: Humbert H Leroy J-F (Eds) Flore de Madagascar et des Comores: plantes vasculaires / publiée sous les auspices du gouvernement général de Madagascar et sous la direction de H.Humbert. Muséum national d’histoire naturelle, Paris, 1–318.

[B10] Pichon M (1949) Les *Carissa* de Madagascar. Mémoires de l’Institut Scientifique de Madagascar, Série B, Biologie Végétale 2: 125–140.

[B11] Richard A (1830) Mémoire sur la famille des Rubiacées, contenant la description générale de cette famille et les caractéres des genres qui la composent. Imprimerie de J. Tatsu, Paris, 1–270., reimpr. (1834) in Mémoires de la Société d’Histoire Naturelle de Paris 5: 81–304.

[B12] Ruhsam M Davis AP (2007) A taxonomic revision of the genus *Flagenium* Baill. (Rubiaceae-Octotropideae). Botanical Journal of the Linnean Society 155: 557–570. doi: 10.1111/j.1095-8339.2007.00714.x

[B13] Schumann K (1891) Rubiaceae. In: Engler A Prantl K (Eds) , Die natürlichen Pflanzenfamilien, vol.4, part 4, Engelmann, Leipzig, 1–56.

[B14] Stearn WT (1957) Achille Richard’s ”Mémoire sur la famille des Rubiacées”. Taxon 6(7): 186–188. doi: 10.2307/1215994

[B15] Tosh J De Block P Davis AP Dessein S Robbrecht E Smets EF (2009) The tribal placement of the monospecific tropical African genus *Petitiocodon* (Rubiaceae) based on molecular data and morphology. Blumea 53(3): 549–565. doi: 10.3767/000651908X607503

